# The workload of web-based consultations with atopic eczema patients at home

**DOI:** 10.1186/1756-0500-3-71

**Published:** 2010-03-12

**Authors:** Thomas RG Schopf, Roald Bolle, Terje Solvoll

**Affiliations:** 1 Norwegian Centre for Integrated Care and Telemedicine, University Hospital of North Norway, Tromsø, Norway; 2 Department of Paediatrics, University Hospital of North Norway, Tromsø, Norway

## Abstract

**Background:**

Atopic eczema is a chronic inflammatory non-contagious skin disease characterised by intensive itch and inflamed skin. Due to its chronic and relapsing course atopic eczema imposes a great burden on affected families. Review articles about home care telemedicine have indicated advantageous effects of home telehealth. However, few studies have investigated how home care telemedicine applications affect the workload of the clinician.

**Methods:**

The use of a web-based counselling system was recorded through computerised logging. The doctor who answered the requests sent via the Internet recorded the amount of time needed for reading and answering 93 consecutive requests.

**Results:**

The time needed by the physician to read and answer a request was less than 5 minutes in 60% of the cases. The doctor spent significantly more time to answer requests that had photographs attached compared to requests without photographs (P = 0.005). The time needed to answer requests received during the winter season (October-March) was significantly longer than the rest of the year (P = 0.023). There was no correlation between the answering time and the age of the patient.

**Conclusions:**

Individual web-based follow-up of atopic eczema patients at home is feasible. The amount of time needed for the doctor to respond to a request from the patient appears to be small. The answering time seems to depend on whether photographs are supplied and also on seasonal variations of disease activity. Since the management of atopic eczema is complex involving many different types of treatments and educational aspects, we expect this type of communication to be useful also to other chronic disease patients requiring close follow-up.

## Background

Atopic eczema (AE) is a chronic inflammatory non-contagious skin disease characterised by intensive itch and inflamed skin lesions with a typical distribution on the skin surface. It is related to other atopic diseases, such as bronchial asthma and hay fever. The incidence of AE in Western countries has increased steadily during the last decades[1]. In Norway it is one of the most common chronic diseases with a cumulative prevalence in children of 20-25%[2]. Due to its chronic and relapsing course with itching, scratching and impaired sleep, AE imposes a great burden on affected families[3]. Management of moderate to severe AE is a therapeutic challenge[4].

The University Hospital of North Norway in Tromsø serves as the main specialist health care provider due to the shortage of both dermatologists and paediatricians in the northernmost health region. Some patients may have to travel up to 400 km by aircraft to see a specialist. Regularly, these patients arrive in the evening the day before they have an appointment at the clinic. Consequently, there is a potential here for telemedicine to improve access to specialist health care, as well as to reduce travel and hotel costs. Studies have shown that store-and-forward technology can be used for remote diagnosis of skin diseases with a level of accuracy comparable with outpatient examinations[5,6]. Several review articles about home care telemedicine have indicated advantageous effects of home telehealth[7,8]. While there is some evidence that patient-physician communication through e-mail and web messaging is time efficient for the doctor, few studies have tried to assess the actual time required to process web messages[9-12]. This report is part of a randomised controlled trial designed to analyse the effects of web-based consultations for parents of children with atopic eczema. One of the conclusions was that such a service is feasible involving a moderate message volume and that the majority of patients were satisfied with the service[13]. Therefore, we conducted an analysis on the time needed by the doctor to process patient messages.

## Methods

Details of study procedures are described in a previous publication[13]. Briefly, patients were consecutively recruited from the paediatric and dermatological outpatient units at the University Hospital of North Norway in Tromsø and Hammerfest County Hospital between August 2005 and September 2006. Children aged 0.5 - 12 years with moderate to severe AE with at least one period of disease activity in the last 12 months were eligible for inclusion. In the intervention group parents were free to send requests via the Internet. They were also allowed to see their primary physician at all times. The Internet communication was accessible briefly after randomisation. In addition to "free writing" parents were asked to use a form showing extent and severity of the eczema. Photographs of affected skin areas could be attached but this was not mandatory. The parents could send requests via the Internet to the specialist ward for general advice at any time or when the eczema flared. There were no limitations concerning the length or frequency of requests provided they were dealing with AE. All parents have given their informed consent.

### Measures

The doctor who answered the requests sent via the Internet recorded the amount of time needed for reading and answering 93 consecutive requests received between January 2007 and June 2008. Messages containing only "thank you" or brief notes were not recorded. Time measurement was done by the use of an ordinary wristwatch with stop-clock functionality. Time was recorded in categories: Less than 5 minutes, 5-10 minutes, and more than 10 minutes. This approach was chosen in order to ease data recording and processing. All chronological data represent the work of one doctor.

### Technical solution

The technical solution used in the project provides functionality that includes a secure messaging system which ensures the patients' privacy while sending photographs and text to their provider via an ordinary web-browser. The images were uploaded directly from the camera leaving no sensitive data on the computer used[13]. The system is based on patient initiated contact, and the specialist usually replied within one working day. One experienced resident in dermatology at the University Hospital of North Norway answered the requests, while two specially trained nurses answered messages when the doctor was on leave or holiday. When an answer was sent, the patient was informed through SMS. There was no system for notifying the health personnel about incoming requests. Daily login was thus generally performed twice a day in order to access requests.

### Statistical analysis

SPSS 15.0 for Windows was used for the statistical analysis. The doctor's time data were analysed to determine if handling requests that had photographs attached was a more time-consuming process. The time of year when the requests were received was also analysed. Requests received from October to March were by definition designated as winter period, the remainder being summer period. Hereby the study period was divided into two equally long time periods. Because the response time data was very skewed (see also table 1), we categorised and applied χ^2^-tests for analysis. All given P values are 2-tailed, P < 0.05 indicates statistical significance.

**Table 1:** Requests per patient

	No requests	No requests with photographs
Patient 1	49	6

Patient 2	11	0

Patient 3	7	3

Patient 4	6	1

Patient 5	5	1

Patient 6	4	0

Patient 7	4	1

Patient 8	2	1

Patient 9	2	0

Patient 10	1	0

Patient 11	1	0

Patient 12	1	0

All	93	13

### Ethics

The Regional Committee for Medical Research Ethics in North Norway and the Data Inspectorate of Norway approved the study protocol.

## Results

During the study period, 12 patients sent a total of 93 requests. While there was an even distribution of requests throughout the period, the number of requests per patient varied, see table 1.

### Workload

The doctor answered 93 consecutive requests received between January 2007 and June 2008. Fifty-six out of the 93 messages (60%) were answered within 5 minutes. Thirty messages (32%) were answered in 5-10 minutes, and 7 messages (8%) in more than 10 minutes. The time needed to answer requests that had photographs attached was significantly longer compared to requests without photographs (P = 0.005). Also the time varied according to the season. The doctor spent significantly more time to answer those 47 requests received in the winter season (October-March) than in the rest of the year (P = 0.023). There was no correlation between the answering time and the age of the patient. Table 2 summarises the distribution of answering times for all patients as well as the patient sending the highest number of requests (Patient 1).

**Table 2:** Distribution of requests (percentage)

	< 5 min	5-10 min	> 10 min
Patient 1	61	33	6

Patient 2-12	59	32	9

All	60	32	8

## Discussion

Several review articles about home care telemedicine have indicated advantageous effects of home telehealth[7,8]. A study by Andreassen et al indicated that 45% of the population wish to use the Internet for communication with their doctor[14]. However, the majority of studies have focused on the transmission of laboratory data, e.g., blood glucose data or spirometry data. Little is known about communication involving "free text" writing via e-mail or web-based systems. In this study we used a secure web-based system to exchange text messages and photographs in the follow-up of eczema patients previously seen at our hospital. Our study showed that follow-up of AE patients through the use of home-based telemedicine is feasible. The amount of time needed for the doctor to respond to a request from the patient appears to be small compared to an ordinary consultation at the hospital, not to mention compared to the time needed by the patient to travel. This is consistent with the findings of Borowitz et al[11]. The time needed for the doctor to read and answer a request was significantly longer during the winter season. This appears to be due to the natural course of AE which often improves in warm sunny weather while the condition worsens in the winter season. The answering time was significantly longer when digital images were attached to the request. Because of the visual nature of dermatology, photographs obviously provide additional information that needs to be processed by the doctor. This result is thus not surprising. Because the management of AE is complex, this type of web-based follow-up seems applicable also to other patients with chronic disease, e.g., epilepsy or psychiatric disorders. We believe transmission of sensitive patient data should be secure and therefore, the use of ordinary e-mail does not appear to be adequate.

There are several limitations of this study. Time measurement was performed by one single physician. We do not know anything about the potential time investment of other doctors. We chose to let one doctor do the work to ensure that a good routine was established first before starting time recordings. Another issue is the self-reporting of time data. Ideally, another independent person should have observed the doctor who handled the requests. A similar method was used in a study investigating time investment of general practitioners in a teledermatology setting[12]. However, the study was performed in a laboratory setting. We considered a similar approach inadequate in our case. We aimed at investigating in a busy and real hospital environment. Furthermore, in a previous study about e-mail consultations Borowitz et al also used self-reported time data obtained by a single physician[11]. The inhomogeneous distribution of the number of requests per patient might introduce bias, e.g., a trend towards shorter answering times for patient 1 because the doctor could refer to earlier responses to the same patient. However, the distribution of the response times did not seem to change when patient 1 was excluded. Furthermore, when dealing with atopic eczema, the inhomogeneous distribution of requests does not seem surprising to us because of the nature of the disease. It can be expected that the need for consultations is variable in different patients depending on the severity of the condition.

## Conclusions

Individual web-based follow-up of AE patients at home is feasible. The amount of time needed for the doctor to respond to a request from the patient appears to be small. The time for an answer seems to depend on whether photographs are supplied and also on seasonal variations of disease activity. Since the management of AE is complex involving many different types of treatments and educational aspects, we expect this type of communication to be useful also to other chronic disease patients requiring close follow-up.

## Competing interests

The authors declare that they have no competing interests.

## Authors' contributions

TRGS had primary responsibility for protocol development, data analysis and writing of the manuscript. RB contributed to protocol development, data analysis and writing of the manuscript. TS had responsibility for the technical solution and contributed to writing of the manuscript. All authors read and approved the final manuscript.
